# Echoes of the Month

**Published:** 1924-03

**Authors:** 


### ECHOES OF THE MONTH.

### THE LONELY "G.P."

"Every day (writes Dr. Frank Layton in an article called 'A Medical Utopia,' which appears in the *English Review*) the G.P. is confronted with the problem of the sick person who has no money. The 'Panel' has taken charge of the wage-earner: the wife, the children, the old mother, the aunts are outside the scheme. But the person too poor to pay a fee is not the only difficulty with which the G.P. is confronted. Day by day he is faced with the problems called up by the fact that he, an individual, has to work as an individual—almost always as an individual. He plays a lone hand. How bleakly lone it is only he can tell. He works alone. He does the whole of his job himself, and by himself. Day after day he sees his patients—alone. In case after case he would like a second opinion; but he cannot get it. The patient cannot afford the fee of a consultant. The doctor very often is not sufficiently in touch with the nearest hospital to get the second opinion there. The G.P. wants modern diagnostic help, laboratory help, bacteriological help. How is he to get it? He just doesn't. He does without. He carries on as well as he can, and in time he dies, a disappointed man. His ideals have come to nothing. His vision of a Real Medical Service has remained a vision—a fantasy—a dream."

### HOW SNORING CAN BE CURED.

The Academy of Medicine (says the Paris correspondent of the *Daily Telegraph*) has just received the good news that snoring can be suppressed, not only painlessly but with benefit to those whose musical slumbers sometimes cause insomnia in others. Dr. Georges Gautier, who has for twenty-seven years been studying the human respiratory system, has concluded that in a large proportion of people, particularly city dwellers, the air passages from the nose to the throat are so small that they materially reduce the supply of fresh air to the lungs, thereby actually shortening life in some cases, and in many others retarding both physical and mental development. Dr. Gautier has therefore perfected a method by which these air passages may be painlessly enlarged. This is achieved by passing from the nostrils to the throat a succession of probes of gradually increasing dimensions. The result, the doctor claims, is not only comparative freedom from such minor annoyances as colds in the head, but better protection from the microbes of diseases and a more efficient cleansing of the blood stream. Deafness, headaches, asthma, and snoring are among the maladies which have already yielded to treatment, of which the doctor computes about 70 per cent. of the population of Paris are in need.

### MOST DANGEROUS AGE FOR CONSUMPTION.

Eighty per cent. of the inhabitants of this country have the germ of consumption in their bodies, but only 20 per cent. of them ever show any sign of the disease, says Dr. H. A. Ellis, assistant physician at Margaret Street Hospital for Consumption. One curious point, for which no satisfactory explanation has been discovered, is that in women the most dangerous age for consumption is between eighteen and twenty-five, and then mostly in young women of Irish stock or descent, and in men about the age of forty-five, when they are inclined to make greater demands on the body than the body can supply. There is no cause for panic if the germ is found after a medical examination, for in nearly all cases resistance can be restored at leisure and the danger removed.

### AVERSION TO SCHOOL A SOUND INSTINCT.

Speaking at Aberdeen on "Education and Nutrition," Dr. Orr said that it is worth while considering whether or not our present educational system is not perhaps too severe and exacting and causes a drain upon the health of a certain proportion of the children attending schools, and whether it would not be better to allow Nature to have a freer hand in the development of the child. The aversion of the healthy boy to the school and lessons is a sound natural instinct. Children spend about five hours a day in school, and have often another hour or two of preparatory work at night. A business man, with a mature mind, would find it difficult to attend in a day five directors' meetings of an hour each and take home a pile of papers in preparation for another five meetings on the following day. For a child with a brain still growing it must be a strain to carry out such a programme conscientiously. The healthy, vigorous boy, of course, does not do it. He finds means of passing part of the time at least otherwise than concentrating on lessons. In this country in many cases, especially in schools in the crowded parts of the towns, we are wasting efficient and valuable educational effort on children who are not in a physical condition to receive the full benefit of it.

### THE ANCIENTS AS DOCTORS.

Dr. Louis Sambon, the eminent authority on malaria, says the *Birmingham Post*, has shattered some of our pride in what we wrongly suppose are purely modern discoveries about this ancient plague. In a pleasantly-learned lecture to the Anglo-Hellenic Society, he told us that there is nothing new, except perhaps some of the methods, in our war on the mosquito. The Romans drained marshes and pools and invented the mosquito net—sure proofs that they more than suspected the part the mosquito played as a disease-carrier. The Romans knew, Dr. Sambon also showed, quite a lot about surgery. He exhibited a dainty set of bronze surgical instruments, probably the property of a Roman army surgeon, and he told of their medical services, their methods of first aid, their army nurses, their field dressings, and bandages—many of them almost exactly like those we were so familiar with only a year or two ago.

### HEALTH OF SHOP WORKERS.

Strongly in favour of requiring that all people having the handling of foodstuffs should be free from tuberculosis, or any kind of skin disease, Dr. A. A. Roberts, a candidate in a municipal by-election at Leeds, declared that he knows of cases in his ward where people suffering from tuberculosis in a form which makes them liable to communicate infection are handling foodstuffs. Dr. J. J. Jervis, the Medical Officer of Health for Leeds, commenting upon the statement, said that at present the authorities could not prevent such a thing. They could not deprive a tradesman of his livelihood, even though he was found to be suffering from tuberculosis, and even if they had the power it would be useless without some method of compensation. But to such people it was pointed out that they could reduce the danger by taking precautions. Particular attention was paid to the tuberculous milkman, but even in his case, it could only be pointed out to him that he was endangering the general community by continuing to handle milk.

### HOW AMERICA DEALS WITH INFLUENZA.

The United States (says the *Manchester Guardian*) seems to be ahead of us in organising methods for dealing with influenza. Two years ago, when the malady threatened to be epidemic, the Health Commissioner of Chicago increased the number of doctors attending the schools and similar institutions and arranged for an emergency corps of doctors and nurses, who were ready for action at all times and in all parts of the city. He also ordered all theatres, churches and street cars to be periodically disinfected, and, realising that cold flats made folk susceptible to the disease, issued a warning that "if he found evidence of a landlord having cut off the heat, and a tenant dying in consequence, he would ask the State Attorney to indict him on a charge of murder."

### LACK OF DOCTORS IN YUGO-SLAVIA.

It is announced at Belgrade that the medical faculty of Zagreb University has been closed indefinitely. The reason given is that the university is in debt for its coal supply to the sum of over one million dinars (nearly £3,000), although this figure must be exaggerated. One serious result of the closing down is that the clinic of the Faculty will also be closed, and the hundred and fifty patients in it awaiting operations will have to be turned away. Belgrade University has only had a medical faculty since the war, and it is not yet possible to complete a course of medicine there, but the Zagreb faculty dates from before the war, and is extremely well fitted. Rapid strides are, however, being made in Belgrade. One enormous difficulty is the absence of any proper serious text-books, and even of a proper nomenclature in the Serbian language, but lecturers of the University are working hard to repair these deficiencies. Still, it must be many years before the home Universities of Yugo-Slavia will be able to produce and maintain a sufficient supply of doctors.

### PAPWORTH HALL SETTLEMENT.

Life at the Papworth Hall Village Settlement (near Cambridge) for the cure of consumptives was described in a lecture, illustrated with films, by Dr. P. C. Varrier-Jones, the Medical Director, at the Royal Institute of Public Health, Russell Square. Dr. Varrier-Jones said that in the cure of tuberculosis the economic factor was the crux. It was no use sending back an infected man to the slums with instructions to take a pint of milk a day and keep his windows open. At the Papworth Settlement it was the family unit which was considered. Cabinet-making, upholstering, and other industries had been established there by which each man did his share of the work and earned a living wage. There were now seventy little children in the Settlement, none of them with any sign of tuberculosis. The men were happy, contented wage-earners, unable to earn as much as an able-bodied man in the outside labour market, for which reason the overhead costs of the industrial section were not made a charge on the proceeds of the furniture and other work sold. They were paid at the rate of 1s. 6d. an hour.

### A DANGER FROM MAH JONG.

Mah Jong, the Chinese game which has made great inroads on the popularity of auction bridge with Americans, has (says the New York correspondent of the *Times*) been discovered to have unsuspected perils. Most of the more elaborate sets of "tiles" with which the game is played are contained in red and black Chinese lacquer boxes. Recently physicians have been puzzled by the number of cases of dermatitis they have been called upon to treat in persons who were devotees of the new craze. Now it has been discovered that in the lacquer is the oil of rhus vernix, and rhus vernix is first cousin to rhus toxicodendron or poison ivy. The inflammation caused by the Mah Jong paraphernalia is so prevalent that it is the subject of two articles in the current issue of the Journal of the American Medical Association by New York and Chicago physicians.

### TREATING "DERBYSHIRE NECK."

A suggestion has been made by Dr. Goodfellow, of Chesterfield, that an attempt should be made to decrease the number of cases of "Derbyshire neck" by treating the public water supply with an infinitesimal quantity of iodine. The *Derbyshire Times* "understands that the authorities are investigating the practicability of the suggestion and are also awaiting further information as to the methods employed at Rochester, U.S.A., and the results obtained. When these are procured we are assured the whole question will be further considered, and it is then quite possible that experiments will be made. The cost of treatment would be small, and certainly the infusion of iodine into the water would not be noticeable.

### A COMMUNITY CHEST.

Striking results are expected from the latest appeal on behalf of the Toronto "Community Chest," the organisers of which set themselves the task of raising £100,000. Previous responses have been excellent. In 1920, from a population of half-a-million, there was collected £47,936; in 1921, £67,932; and in 1922, £77,860. These results were obtained at a cost of only 5 per cent. of the total raised, a vast economy on the making of individual appeals. A "Community Chest" is an organisation of which we rarely hear in this country, and for which certainly we have no equivalent. It is a co-operative organisation charged with the duties of raising necessary funds for existing social bodies, the intention being to obtain all the money required for all sorts of purposes, every year, in one big, well-advertised, well-directed campaign.

